# Treatment of Burning Mouth Syndrome With Amisulpride

**DOI:** 10.4021/jocmr972w

**Published:** 2012-05-15

**Authors:** Carmen Rodriguez-Cerdeira, Elena Sanchez-Blanco

**Affiliations:** a Dermatology Department, CHUVI and University of Vigo, Vigo, Spain; b University of Vigo, Vigo, Spain

**Keywords:** Burning mouth syndrome, Amisulpride, Women

## Abstract

**Background:**

Burning mouth syndrome (BMS) is a frequently occurring disease characterized by a burning or painful sensation in the tongue and/or other oral sites without clinical mucosal abnormalities or lesions. Its etiopathology is unknown, although local, systemic, and psychological factors have been associated with BMS. The syndrome is multifactorial, and its management remains unsatisfactory. The purpose of this study was to obtain preliminary data regarding the efficacy and tolerability of amisulpride in BMS treatment.

**Methods:**

The subjects were treated with amisulpride (50 mg/day) for 24 weeks. Efficacy assessment included a visual analogue scale (VAS) for pain intensity, the Hamilton Rating Scale for Depression (HAM-D), the Hamilton Rating Scale for Anxiety (HASM-A), and the Clinical Global Impression Scale-Efficacy Index (CGI-EI).

**Results:**

The treatment regimens resulted in a significant improvement in burning mouth symptoms from baseline at week 24, as indicated by the quantitative mean illness duration VAS score, HAM-D, and HAM-A. Amisulpride appears to be effective and patients show a rapid response to treatment. No serious adverse effects were encountered in these patients.

**Conclusions:**

Amisulpride is effective and well tolerated as a short-term treatment. It is particularly efficacious at the start of treatment and has shorter response latency. Double-blind placebo-controlled trials are needed for further assessment of the efficacy of amisulpride in BMS treatment.

## Introduction

Primary burning mouth syndrome (BMS) is a chronic, idiopathic intraoral mucosal pain condition that is not accompanied by clinical lesions or systemic disease. Whether this condition should be referred to as a disease, disorder, or syndrome is uncertain, but data to justify any change in taxonomy are insufficient at present. BMS is often accompanied by xerostomia and taste disturbances. A neuropathological basis for BMS has recently been proposed, suggesting that it could be regarded as an oral dysesthesia or painful neuropathy. However, our incomplete understanding of the epidemiology, etiology, and pathophysiology of BMS, and the lack of any diagnostic criteria, are barriers to critical investigation and the selection of effective treatments [1]. There is only limited evidence to guide clinicians in the management of patients with BMS. The estimated prevalence of BMS reported in recent studies ranges between 0.7% and 4.6% of the general population; most cases occur in postmenopausal women. The rate of occurrence of this disorder in women is 20% higher than that in men [2]. The most prevalent comorbidity is a major depressive episode. No other psychiatric disorders have been associated with BMS in comparison with healthy people [3, 4].

Amisulpride is a selective dopamine antagonist. It has a high affinity for D_2_ (Ki = 2.8 nM) and D_3_ (Ki = 3.2 nM) dopaminergic receptors. Dosage ranges from 200 to 1200 mg/day. Lower doses (less than 50 mg) preferentially block D_2_ autoreceptors, which control the synthesis and release of dopamine. This results in an increase in dopaminergic transmission, which is hypothesized to cause a reduction in both depressive and negative symptoms. Higher doses of the drug block the postsynaptic dopamine receptors, resulting in an improvement in symptoms of psychosis. It is an atypical antipsychotic drug and has a low incidence of extrapyramidal side effects [5].

Although substantial evidence indicates the efficacy of some antidepressants in treating psychogenic pain and somatoform disorders, very few studies have investigated their possible therapeutic action in cases of BMS. The aim of this study was to demonstrate the efficacy and tolerability of amisulpride in a group of patients with BMS.

## Materials and Methods

Patients for this study were recruited at the net of the hospitals of the National Galician Health Service in the Northwestern of Spain. Twenty-four subjects with BMS were screened consecutively for inclusion in this study, but only 22 patients, all female, gave their written consent and participated. All patients met the DSM-IV (Diagnostic and Statistical Manual of Mental Disorders, 4th edition) diagnostic criteria for BMS.

Depressive symptoms were evaluated according to the Hamilton Rating Scale for Depression (HAM-D); anxiety symptoms were evaluated using the Hamilton Rating Scale for Anxiety (HAM-A); and the clinical efficacy and tolerability of the treatment were assessed using the Clinical Global Impressions scale (CCG). The rating scales were administered at baseline and every 2 weeks until week 8 and then every 4 weeks until 24 weeks. Diagnostic assessment was carried out using the Diagnostic Interview Schedule-Revised (DIS-R, a highly structured interview that carefully specifies the questions that the interviewer must ask to make a diagnosis). Patients were excluded if they had a severe illness, were pregnant or lactating, had a history of breast or genitourinary cancer, or had an allergy or intolerance to amisulpride. Patients who met the DSM-IV criteria for psychotic disorders or concurrent depression were also excluded from the study. None of the patients had taken any psychoactive drug for at least 4 weeks before the time of admission, and no concomitant psychotropic or psychotherapeutic treatment was permitted for the duration of study. Other oral diseases/disorders were ruled out prior to inclusion in the study.

At the time of recruitment, each patient was treated with 50 mg of amisulpride. The treatment was continued for 6 months at the same dose. Failure to comply with treatment (missing more than 2 consecutive doses of the drug) and withdrawal of participant’s consent were the criteria for withdrawal from the study.

### Clinical assessment

The patients were asked to indicate the mean pain intensity during the week preceding the consultation by scoring on a vertical 10-cm visual analogue scale (VAS).

Depressive symptoms were evaluated according to the HAM-D scale; anxiety symptoms, using HASM-A; and clinical efficacy and tolerability of the treatment, using CGI-EI. The rating scales were administered at baseline and every 2 weeks until week 8 and every 4 weeks until the end of the study (24 weeks) by a trained psychologist.

### Statistical procedures

Analysis of variance (ANOVA) was used to test for comparability of treatment by using continuous variables such as index age, age onset, and baseline scores for the VAS, HAM-D, HAM- A, and CGI-Efficacy Index (EI).

A comparative qualitative evaluation of treatment response was also performed by calculating the percentage of responses in each group. The software used for the analysis was the Statistical Package for the Social Sciences (SPSS), version 18.0.1) (SPSS Inc, Chicago, IL).

## Results

Twenty-two patients were recruited and assigned to receive amisulpride. One patient was excluded for the lack of compliance and another refused to continue the study because of side effects. Ultimately, 20 patients were included. A summary of demographic information, including gender, index age, age at onset of BMS, and illness duration is shown in Table 1.

**Table 1:** Patient’s Characteristics

Gender (female)	Age	Age at onset	Illness duration at our first time (months)	Previous treatments
Female	56	54	36	Fluoxetine
Female	67	65	34	Paroxetine
Female	65	65	37	Fluoxetine,
Female	73	72	24	Paroxetine
Female	49	48	34	Paroxetine
Female	67	66	18	Paroxetine
Female	58	57	24	Paroxetine
Female	54	53	27	Sertraline
Female	68	66	33	Sertraline
Female	60	59	18	Paroxetine
Female	71	70	25	Sertraline
Female	58	56	34	Fluoxetine
Female	64	63	12	Fluoxetine
Female	71	69	8	Paroxetine
Female	61	61	12	Paroxetine
Female	45	43	14	Paroxetine
Female	54	53	12	Paroxetine
Female	56	53	16	Fluoxetine
Female	65	64	33	Fluoxetine
Female	57	56	24	Fluoxetine
Mean ± SD	60.95 ± 7.57	59.65 ± 7.70	2.75 ± 9.48	Fluoxetine

These variables did not differ significantly with respect to the total scores at baseline (VAS, HAM-A, HAM-D, and CGI-EI).

The amisulpride treatment regimen resulted in a significant improvement in BMS from the baseline at week 6 (Table 2), as demonstrated by VAS total scores at the end of the study. A large improvement was also observed in the final HMA-D and HMA-A total scores from baseline to the end of week 8 (Table 3). Both the severity of illness (CGI-S) and improvement (CGI-EI) scales showed steady improvement over 8 weeks. The mean ± SD CGI-EI scores were 4.2 ± 1.2 at baseline and 2.5 ± 0.9 at week 8 and remained more or less constant until week 24.

**Table 2:** Rating VAS, HMA-A, HAM-D Scores (mean ± SD) During Treatment With Amisulpride

Scale	VAS	HAM-A	HAM-D
Baseline	8.35 ± 0.67	21.10 ± 1.91	20.80 ± 1.47
Week 2	7.0 ± 0.56	15.20 ± 1.36	14.05 ± 1.93
Week 4	5.85 ± 0.87	3.15 ± 1.69	10.80 ± 2.41
Week 6	3.35 ± 0.58	7.90 ± 1.07	8.60 ± 0.59
Week 8	1.15 ± 0.93	4.85 ± 1.18	6.80 ± 0.76
Week 12	1.15 ± 0.81	6.65 ± 0.67	4.70 ± 1.12
Week 16	1.15 ± 0.87	6.45 ± 0.51	4.70 ± 1.03
Week 20	1.05 ± 0.88	6.60 ± 0.75	4.80 ± 1.19
Week 24	1.05 ± 0.87	6.55 ± 0.68	4.68 ± 0.98

VAS: Visual Analogue Scale; HAM-A: Hamilton Rate Scale for Anxiety; HAM-D: Hamilton Rate Scale for Depression.

**Table 3:** Cumulative Proportion of Responders at Weeks 2, 4, 6, 8, 12,16, 20 and 24 During Treatment With Amisulpride (NP = 20)

Time	Number of patients	%
Week 2	6	30
Week 4	8	43
Week 6	17	85
Week 8	20	100
Week 12	20	100
Week 16	20	100
Week 20	20	100
Week 24	20	100

NP: Number of the patients.

VAS, HAM-A, and HAM-D mean scores decreased significantly from baseline to week 2 (Table 2), indicating the speed of action of amisulpride. These scales also show that this improvement increased significantly until week 6. The percentage of response at week 8 was 100% (Table 3) and remained at this level through week 24. No serious adverse effects were reported. However, the most frequently encountered side effects were as follows: insomnia in 4 patients, tremor in 3 patients, headache in 2 patients, nausea/dyspepsia and sedation in other patient. The occurrence of these side effects is shown in Table 4.

**Table 4:** Side Effects

	Number	%
Nausea/dyspepsia	1	0
Sedation	1	0
Dry mouth	0	7
Constipation	0	13
Insomnia	4	13
Anxiety	2	20
Tremor	3	7
Asthenia	0	0
Headache	3	13
No side effects	6	40

Number of patients = 20.

## Discussion

We carried out a review of the articles and clinical case studies published in the literature between 2001 and 2011, describing different pharmacological options for the treatment of BMS. To our knowledge, this is the first systematic study of amisulpride in the treatment of BMS by using established diagnostic criteria and rating scales over a long period. The data relating to the efficacy and safety of each of the drugs used in the different studies have been reviewed. However, the clinical trials conducted to date are not particularly robust. Most of them are open-label uncontrolled studies involving small patient groups and short time periods. Furthermore, the expression of results is heterogeneous [6, 7].

Although effective therapies have been identified in specific cases, a treatment modality that offers efficacy in most cases of BMS has not yet been established. Further insights into the psychopathological mechanisms of BMS and establishment of differential diagnostic criteria are needed in order to develop drugs with improved efficacy and safety profiles for the treatment of BMS [8-10].

This study showed that amisulpride is efficacious in the treatment of BMS, according to the mean reduction in VAS, DAM-D, and HAM-A scores. With regard to the qualitative evaluation of clinical response, the percentage of responders was high. No significant differences were found between demographic and clinical variables. Moreover, the demographic and clinical features of our sample were similar to those of other studies, except with regard to sex; in our study, all patients were female [11-13].

Regarding treatment duration, analysis of the studies showed that a long-term treatment (over months) was not necessarily more effective [1, 14]. However, we believe that investigation of the long-term duration of treatment is important in order to generate more reliable results in terms of both clinical outcomes and the possible adverse effects that may occur during the course of extended treatment. Many of the studies reviewed did not follow-up the patient [15]. In this respect, adequate follow-up is needed to guarantee the efficacy of amisulpride therapy.

Three limitations of the study need to be noted. First, this study was performed on an open-label basis and without placebo control. It should be noted that placebo response rates of up to 50% have been reported in previous studies, and only a double-blind, placebo-controlled randomized trial can determine the true therapeutic profile of amisulpride in the treatment of BMS. Second, whether treatment response is similar in male patients was not determined. Third, the efficacy and tolerability of amisulpride have been tested in short-term observations, but they also need to be investigated in long-term observations.

### Conclusion

Amisulpride is efficacious and well tolerated in the short-term treatment of BMS. It is associated with good compliance in the first week of treatment and short response latency, and is especially useful at the beginning BMS therapy. However, further studies of this potentially important condition are needed and the potential benefits of amisulpride should be confirmed under placebo-controlled conditions. In this sense, it would be interesting to homogenize criteria for expressing the results obtained.
